# Using co-design to identify healthcare priorities for patients with incurable head and neck cancer

**DOI:** 10.1186/s12913-025-13993-y

**Published:** 2026-01-12

**Authors:** A. Achinanya, V. Bryant, S. Payne, L. Sharp, M. Harrison, D. Hamilton, C. M. Kim, M. Offerman, J. M. Patterson, C. R. Mayland

**Affiliations:** 1 Division of Clinical Medicine, School of Medicine & Population Health, University of Sheffield, Sheffield, UK; 2 CHANGE PPI group, Sunderland, UK; 3 Lancaster University, Lancaster, UK; 4 Newcastle University, Newcastle, UK; 5 Newcastle Hospitals NHS Foundation Trust, Newcastle, UK; 6 Erasmus University Medical Center, Rotterdam, Netherlands; 7 University of Liverpool, Liverpool, UK; 8 Sheffield Teaching Hospitals NHS Foundation Trust, Sheffield, UK

**Keywords:** Incurable head and neck cancer, Co-design, Patient and public involvement, Interventions, Qualitative research

## Abstract

**Background:**

Patients with incurable head and neck cancer (HNC) face complex care pathways, significant symptom burdens and psychosocial challenges. The complexity of symptoms, disease trajectory and the centralised, but often inequitable, services frequently lead to the patients’ and caregivers’ needs for support and care not being fully met. To address this gap, this study adopts a co-design approach, where patients, caregivers, and professionals collaborate to develop solutions that address service issues, aligning with the needs and priorities of both patients and caregivers.

**Methods:**

This qualitative exploration of co-design processes involved patients, caregivers, and healthcare professionals (HCPs) participating in one online and two in-person multi-stakeholder co-design workshops in Sheffield, UK. Patient vignettes were developed to illustrate typical care journeys and ‘stress points’ in service interactions. These vignettes were shared with 13 participants, including patients with lived experience of head and neck cancer, family caregivers, specialist nurses, and allied HCPs, to identify areas for improvement and co-develop potential solutions using prioritisation activities, group concept mapping, and facilitated group discussions.

**Results:**

During the first in-person workshop, co-design participants (co-designers) identified and prioritised critical stress points in the care pathway, including a lack of support in caregivers’ preparedness and challenges navigating healthcare systems (specifically contacting the clinical team). Using these findings, the co-designers proposed various solutions, including introducing a single point of contact (care navigator) or a printed version of a personalised ‘roadmap’ of services, instituting a multidisciplinary discharge planning process to aid transitions to home care and implementing a dedicated 24-hour helpline staffed by knowledgeable personnel (HNC specialist staff) to provide patients with information.

**Conclusion:**

The co-design workshops have developed practical, user-informed intervention solutions to address the specific navigation challenges faced by people with incurable HNC. While the interventions developed are relevant in many ways to the broader HNC care pathway, they are particularly relevant to the complex needs of this group and are now guiding the next phase of interventions for improving patient-centred services.

## Introduction

Head and neck cancer (HNC) comprises a diverse group of malignancies arising in the oral cavity, pharynx, larynx, nasal cavity and sinuses, and salivary glands. This heterogeneity contributes to the complex and varied care needs experienced by people affected by these cancers [1, 2]. It is the seventh most common cancer diagnosed globally, with most patients presenting with advanced disease, which can often be incurable [3]. HNC is classified as ‘incurable’ when radical surgery or full-dose radiotherapy cannot be offered due to the extent of the disease, previous treatments, or metastases [4, 5]. People living with incurable HNC often face a challenging journey characterised by complex symptoms [6–8]. The disease and its treatment have a profound impact on vital physical functions, such as breathing, speech, and swallowing, and significantly affect daily living and quality of life, highlighting the need for specialised care strategies [8, 9]. The five-year survival rate ranges from 35% to 70%, depending on the tumour stage and location [10, 11]. Given the particularly poor prognosis for those with incurable disease, it is crucial to provide support at diagnosis and throughout the illness for this group. Understanding the experiences of this vulnerable population is essential for providing a comprehensive, person-centred approach to addressing their most pressing needs [12].

Co-design in healthcare puts patients and caregivers at the heart of service redevelopment ensuring that solutions are more likely to mirror people’s lived experiences rather than being based on professional judgements and assumptions [13]. For HNC patients, where complex symptoms exist and significantly impact daily life [6, 14], the co-design of services should be paramount. Existing HNC research shows that co-designed programmes improve nutrition support, symptom management, and follow-up care by blending service user experience and clinical expertise to find viable solutions [1, 15, 16]. When patients, caregivers, and healthcare professionals (HCPs) work in partnership to build solutions, services are not only more likely to be feasible for service providers and acceptable to HCPs but also genuinely responsive to the multifaceted needs of patients [9, 17, 18].

**Fig. 1:**
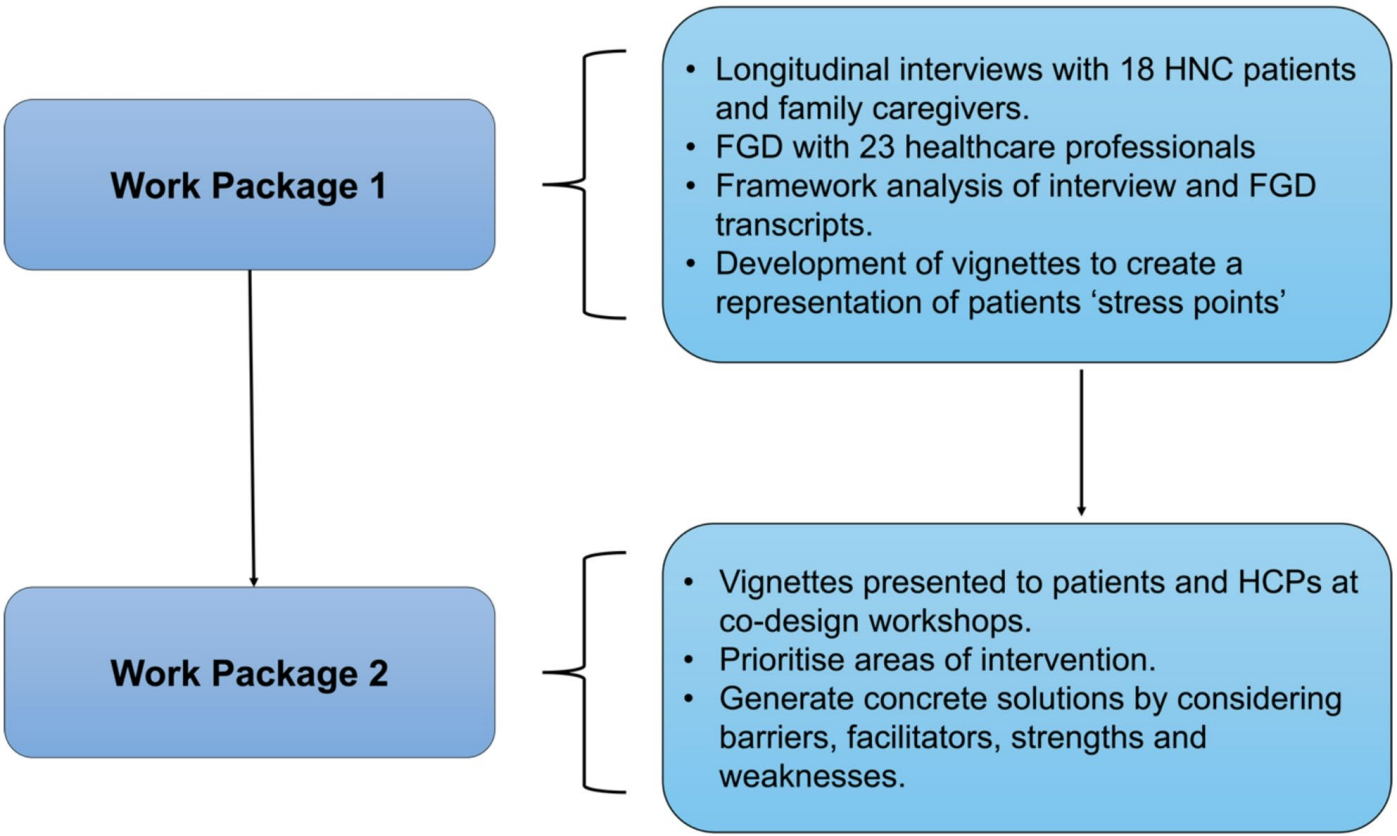
Sequential method employed for the co-design process

This paper is based on a larger sequential qualitative study consisting of two work packages (WP): (1) longitudinal interviews with patients living with incurable HNC cancer and their family caregivers and focus group discussions (FGD) involving HCPs [19], and (2) a co-design process with patients with lived experience of HNC, caregivers, HCPs and the research team members to collaboratively design interventions that would address the needs (Fig. 1). The serial interviews, focus group discussions (FGDs), and framework analysis of the data have been previously described and reported elsewhere [20].

This paper reports on a co-design process (WP2) which aimed to identify priority areas for improvement and co-develop potential solutions in healthcare service delivery, led by patients, caregivers and HCPs in response to the identified challenges faced by patients with incurable HNC. The insights gained through this collaborative approach will help shape interventions that address some specific challenges faced by those living with incurable HNC and their caregivers.

## Methods

This study employed the initial four stages of an established seven-stage iterative co-design process (Table 1), with the additional stages representing future work [17].

**Table 1:** The 7-stage iterative co-design process

Stages	Steps
Stage 1	This involves gathering the evidence from previous research and expert advice to understand what the intervention should include.
Stage 2	This involves checking with stakeholders/co-designers to ensure it is relevant to their needs.
Stage 3	This involves working with co-designers to develop the ideas into early sketches of how the intervention might look or work.
Stage 4	This involves refining the best ideas into a detailed plan that outlines what the intervention will include and how it will function.
Stage 5	This stage leads to the development of a working prototype
Stage 6	This stage involves usability testing to identify any design issues.
Stage 7	This stage focuses on using the feedback to improve the design and content.

We provide a brief description of the methods and outcomes from the sequential interviews [19] and then focus on the methods and results from the co-design process.

### Stage 1: Gathering the evidence (brief description of WP1)

We conducted a qualitative longitudinal study using semi-structured one-on-one interviews with patients living with incurable HNC to understand their perspectives on healthcare experiences [19]. Picker’s principles of patient-centred care [21] served as a framework to guide the development of the interview questions and the analysis of participants’ responses.

Figure 1: Sequential method employed for the co-design process.

#### Recruitment (for WP1)

The study was conducted across three HNC centres based in Northern England. Adult patients (≥ 18 years) with incurable HNC and able to provide informed consent were approached for participation. Patients were asked to nominate a caregiver, either to support them during their interviews (if they wished) or to participate as a ‘proxy’ if they (the patient) became too unwell or died. HCPs involved in incurable HNC care were invited to participate in focus group discussions (FGD). This included oncologists, specialist nurses, General Practitioners (GP), community-based nurses, pharmacists, dietitians, speech and language therapists (SALT) and palliative care practitioners.

#### Data collection and analysis (of WP1)

Trained qualitative researchers (MH and CRM) conducted interviews with consenting patients and their caregivers between May 2023 and July 2024. All interviews were conducted either face-to-face or by phone, audio-recorded, transcribed, and anonymised. Another qualitative researcher (AA) analysed the data using the framework approach [22], with the initial framework based on Picker’s principles of patient-centred care. NVivo was used to support the coding and organisation of data [23].

### Outputs (from WP1)

#### Vignettes

The two major themes from the interviews and FGD (namely, systemic variability of healthcare delivery and difficulties navigating the healthcare system) were used to create two vignettes that represent the ‘typical challenges’ faced by these patients. Vignettes are “*incomplete short stories (narrative accounts) that are written to represent*,* in a less complicated way*,* real-life situations to enable discussion*,* and perhaps resolutions*,* to problems where there are multiple solutions*” (p. 20) [24]. A collection of the challenges highlighted by the study participants and caregivers was used to create two short, tangible narratives, each representing a fictional patient (persona) with incurable HNC, that depicted realistic care scenarios [25] to serve as discussion prompts in the co-design workshops.

#### Designing the vignettes

In designing our vignettes (see Table 2), we considered and prioritised several elements: presentation, length, settings, terminology, and open-ended questioning [26]. ***Presentation***: Our vignettes featured visual representations of the fictional personas, such as an older male patient named John and a middle-aged female caregiver named Martha. Research shows that images included in vignettes provide rich, clearly understandable information reflecting real-life situations [26, 27]. ***Length***: We briefly described different issues raised, such as challenges with swallowing medications, in the vignettes to ensure they engaged the co-design participants’ attention and encouraged responses [28]. ***Scene setting and Terminology***: The selected settings, such as a 72-year-old retired widower at home in a terraced house or sitting in an armchair, were intended to be representative of the patient group. To help readability, lay language was used to describe terms like ‘tracheostomy’ and PEG-tube’ as ‘surgically inserted breathing tube’ and ‘surgically inserted feeding tube’. ***Open-ended questioning***: At the end of the vignettes, we posed questions such as, ‘What challenges do you see in supporting family members caring for someone with cancer?’ which were used to guide the discussion. The research team anticipated that participants would respond orally to these questions during the workshops, allowing prompting with follow-up questions.

**Table 2:** Overview of the vignette framework for one persona

Elements	Characteristics	Descriptors
Person with incurable HNC	Sex	Male (named John*)
	Age	72
	Characteristics	Retired Factory worker
	Relationship	Widower with 1 daughter (named Mary*)
	Diagnosis	Metastatic Tonsil Cancer
Setting	Home	Sits in armchair, Lives in a small, terraced house in a town in the North of England.
	Transport to clinic	Daughter usually helps with transportation to and from hospital visits
	Waiting time	Diagnosed after waiting for 12–18 months for an appointment. Describes the NHS^a^ as a “merry-go-round”
Services included	Clinical team	Contacting the team is via their daughter or via emergency services telephone line (called 999), no primary point of contact
	SALT^b^	Medication swallowing difficulties described by daughter
	GP^c^ & District Nurses	Challenges with medication from GP and having to justify requests that come from the hospice team. Community nurses unable to perform certain tasks and don’t make regular visits.

*The names John and Mary are pseudonyms, ^a^ National Health Service, ^b^ Speech and Language Therapist, ^c^ General Practitioners

Each vignette incorporated challenges faced by patients and caregivers. For instance, the first vignette featured ‘John’, a retired, widowed patient who had challenges contacting the clinical team, accessing specific and important medications, and swallowing difficulties. The second vignette highlighted Martha, an employed family caregiver with a child; Martha faced challenges in managing the emotional and physical demands of providing home care. Additional contextual information was provided for each persona, including family dynamics, employment status, diagnosis, health literacy, and interactions with services. These vignettes drew on genuine quotes and situations found in the qualitative patient interview data (with identifying details changed), aiming to add credibility and relevance. The vignettes served to personify the problems, making them relatable to all co-design attendees regardless of their background, and helped spark rich discussions.

The vignettes were written by the lead author in English, and analytic validation was conducted by all other authors to ensure consistency in interpretation. The team piloted the vignettes with stakeholders from our patient and public involvement (PPI) group to gather their feedback on the relevance and clarity of the vignettes. As our vignettes contained sensitive narratives, piloting provided an opportunity to gauge if there might be any emotional reactions. The feedback was positive, with most PPI representatives perceiving they could relate to the experiences. Although no content changes were needed after the pilot, the team decided to include a ‘content warning’ to the co-designers before sharing the vignettes with them. The team also ensured a safe space was provided by offering a separate quiet room where participants could take breaks, process their emotions, and receive appropriate support if they became overwhelmed.

### Co-design workshop (WP2)

The NIHR’s guidance on co-producing research highlights four core principles: sharing power, including all perspectives, building and maintaining relationships, and respecting and valuing all knowledge and skills, to ensure genuine, equitable partnerships throughout the study lifecycle [29]. It emphasises clear communication, agreed-upon roles, and adequate support and resources, ensuring that public contributors and researchers work together from design through delivery and evaluation. Drawing on these principles, we implemented co-design workshops in our study as follows:

#### Recruitment

Patients or family members who participated in the qualitative interviews (WP1) and consented to be contacted again were invited; however, none decided to participate. Therefore, patient co-designers were recruited through the networks of the PPI members. We invited HCPs and others involved in the care of HNC patients, such as service managers, to participate in the co-design workshops. Our goal was to have a broadly equal mix of patients/caregivers and clinical experts in each co-design workshop group, providing varied perspectives. Overall, 13 stakeholders were recruited to the co-design process (Table 3).

#### Participants

At each workshop, the co-designers were 5 adults (three patients and two caregivers) with lived experience of HNC, 4 HCPs, and 4 members of the research team (2 clinical academics acting as facilitators, 1 health psychologist, and 1 research associate), as well as an academic secretary to take notes on participants’ comments and ideas. The workshops were held in a hospital meeting room and lasted approximately 4 h, including refreshment breaks. Patients and caregivers were reimbursed for their expenses and compensated for their time.

**Table 3:** Demographic characteristics of total co-design participants

Characteristics	Lived Experience (*n* = 7)	HCP (*n* = 6)
**Age** < 65 ≥ 65	1 6	6 -
**Gender** Male Female	5 2	- 6
**Ethnicity** White British/ White European Asian or Asian British	6 1	5 1
**Living Situation** Lives alone With spouse/partner	1 6	- 6
**Experience of HNC** Patient Carer	5 2	N/A -
**Current area of Work** Physician/Surgeon Nurse Specialist Allied Health Professional Pharmacist Others ^a^	N/A	- 3 1 1 1
**Attendance** Workshop 1 Workshop 2	5 5	4 4

^a^ refers to other professionals who work with HNC patients, such as service managers. Footnote: This table represents the total number of unique participants across the co-design workshops. As not all individuals attended all sessions, per-workshop attendance differs from the overall totals reported here

### Stage 2: Checking with stakeholders/co-designers (pre-workshop online session)

An online pre-workshop session was held for participants to familiarise themselves with each other and align their expectations and preferences regarding the co-design process. This session aimed to provide all participants with a foundational understanding of the principles and practices involved. To address power imbalances and create a safe space for open dialogue, strategies for navigating conversations were shared. These included using first names instead of professional titles, and HCPs refraining from wearing their uniforms during the sessions to minimise the potential power imbalances that might arise.

### Stage 3: Working with co-designers to develop ideas

#### Procedure

The research team employed a semi-structured agenda to guide the co-design session. Co-designers were welcomed, (re-)introduced to each other, informed about the workshop’s purpose, housekeeping measures, and the plan in case they became distressed during the session. Furthermore, one facilitator, a trained health psychologist (S.P.), was available to recognise signs of distress and provide appropriate support to participants throughout the sessions.

Together, the group established ground rules for the session, including respect, listening to all voices, maintaining confidentiality, avoiding medical jargon, and understanding that there are no “wrong” ideas. A short icebreaker activity was conducted to foster a friendly atmosphere and keep the conversations light at the outset. The workshop was audio-recorded with participants’ consent.

The patient and family carer vignettes were then introduced, and all co-designers were asked to discuss them in pairs, supported by roaming research team members and a lead facilitator. In the workshops, ‘stress points’ were defined as moments in the vignette when patients or caregivers experienced heightened uncertainty, distress, or difficulty along their care pathway. Facilitators guided participants through a structured deconstruction of the vignette using prompts such as: ‘What challenges do you see in this story?” ‘What challenges do you see around supporting family members who are caring for someone with HNC?’ and ‘What barriers or unmet needs arise at this moment?’ Participants used sticky notes to jot down the issues they recognised, drawing on emotional reactions, personal lived experience, and professional observations of similar cases.

In a whole-group discussion, each pair shared their insights, discussing topics such as health literacy of family caregivers and the need for HCPs to understand the profile of the carer and the nature of their support network. Co-designers discussed the key principles of patient-centred care and ensuring timely and coordinated care. The group reflected on how these principles were addressed (or not addressed) in the vignettes. In examining the stories, the co-designers suggested what would be required to achieve patient-centred care, such as better communication among healthcare services or improved carer information, based on variations in literacy levels and carer preferences. After discussing the issues, the main stress points that had been raised were information needs and preferences, access to medications, family preparedness for caregiving, challenges in emergency situations, and navigating the system (regarding contact with clinical teams).

These stress points were then placed as pictorial descriptors on the walls in the room for participants to vote. Each participant was given three sets of dot voting [30] stickers: red (don’t need to do), orange (might need to do), and green (must do) to prioritise which stress points were most important to address. The stress points ranked the highest were ‘navigating the system’ and ‘family preparedness for caregiving.’ A brainstorming session was conducted to generate initial ideas for potential solutions or interventions.

#### Analysis

At the end of the first workshop, the research team gathered the unique ideas generated by each group. These include detailed notes, individual group notes, and ‘post-it’ notes produced by participants. We analysed the workshop notes and materials using descriptive analysis, focusing on the stress points that participants collectively prioritised, as well as the specific intervention ideas or solutions proposed.

#### Output

Participants generated five intervention ideas (Table 4) to facilitate healthcare navigation for people with incurable HNC and their caregivers.

**Table 4:** Intervention ideas generated in workshop 1

Title	Details	What is hoped to achieve
Cancer Support Navigator	A dedicated point of contact, assigned at diagnosis, via trained staff, to handle practical needs, check-ins, prescription follow-up, and proactive outreach.	This role would foster trust in the service by simplifying care navigation and reducing uncertainty, as many individuals might hesitate to initiate contact themselves.
Visual care team ‘Roadmap’	A simple A4 magnetic sheet to put on the fridge that includes photos, names, roles, and direct numbers of key providers (e.g., speech therapist, CNS) that can be referred to in the home.	This would help patients and caregivers easily recognise who they need to contact and for what service.
Customisable reminders and communications pack	A unified reminder system (SMS, email, call) for appointments, medications, and activities, plus a modular booklet/app listing who to call for specific issues and FAQs to support self-management.	This would enhance communication using the patients preferred communication method and can provide guidance on when to reach out, depending on patient’s needs.
Voice enabled digital assistant	A hands-free device or app linked to GP and NHS 111, delivering medication alerts, hospital maps, and information about local resources	This would be vital for patients with speech or dexterity challenges and the inclusion of the links to support services and support group, could provide easy access to relevant resources.
Culturally tailored information ecosystem	Adapting proven international models of care from different cancer sites, assess their potential applicability for the UK system to enhance local care practices.	This would ensure an inclusive, context-appropriate guidance and prepare a transition framework for palliative care which would be culturally appropriate.

All these ideas presuppose a multidisciplinary team (MDT) model of care planning, which extends beyond tertiary services to involve the community and the voluntary sector. Participants repeatedly emphasised the importance of involving the patient and caregiver in MDT planning discussions to ensure that the care plan was transparent and that the decision-making process was shared.

### Stage 4: Refining best ideas (co-design workshop 2)

#### Procedure

The focus shifted to refining the highest-priority ideas and further developing early sketches of the proposed interventions. In the first activity, participants refined their ideas using outputs from the first workshop and facilitated discussions. They then dot-voted (as in Workshop 1) to prioritise interventions. The top three were the support roadmap for every patient at discharge (a simple diagram of key contacts), the discharge team meeting process, and a 24-hour helpline.

Co-designers were divided into three small groups, each consisting of three individuals: a patient or caregiver, a healthcare professional and a member of the research team, who also acted as the facilitator. Facilitators guided the structure and timing of the activities. To explore each potential intervention, the ideation template designed by Kim et al. (2024) was used for group concept mapping. Following the template, which contains questions such as ‘how does it work?’, ‘what problem does it solve?’, and ‘what needs to be done to execute the plan?’ [31], ideas were outlined, and further discussion took place regarding feasibility, acceptability, anticipated challenges and mitigators.

Towards the end, all participants reconvened in a single group to review proposed interventions, assess feasibility (“what and when?”), and identify potential challenges to implementation, proposing ways to mitigate them through evaluations. Facilitators summarised the proposed solutions and asked participants if their summary reflected their ideas and whether anything was missed, ensuring member checking was done, albeit informally. Any discrepancies were discussed and resolved. At the end of the workshop, the facilitators summarised the agreed-upon priorities and next steps, thanked participants, and acknowledged their contributions.

#### Analysis

**Fig. 2:**
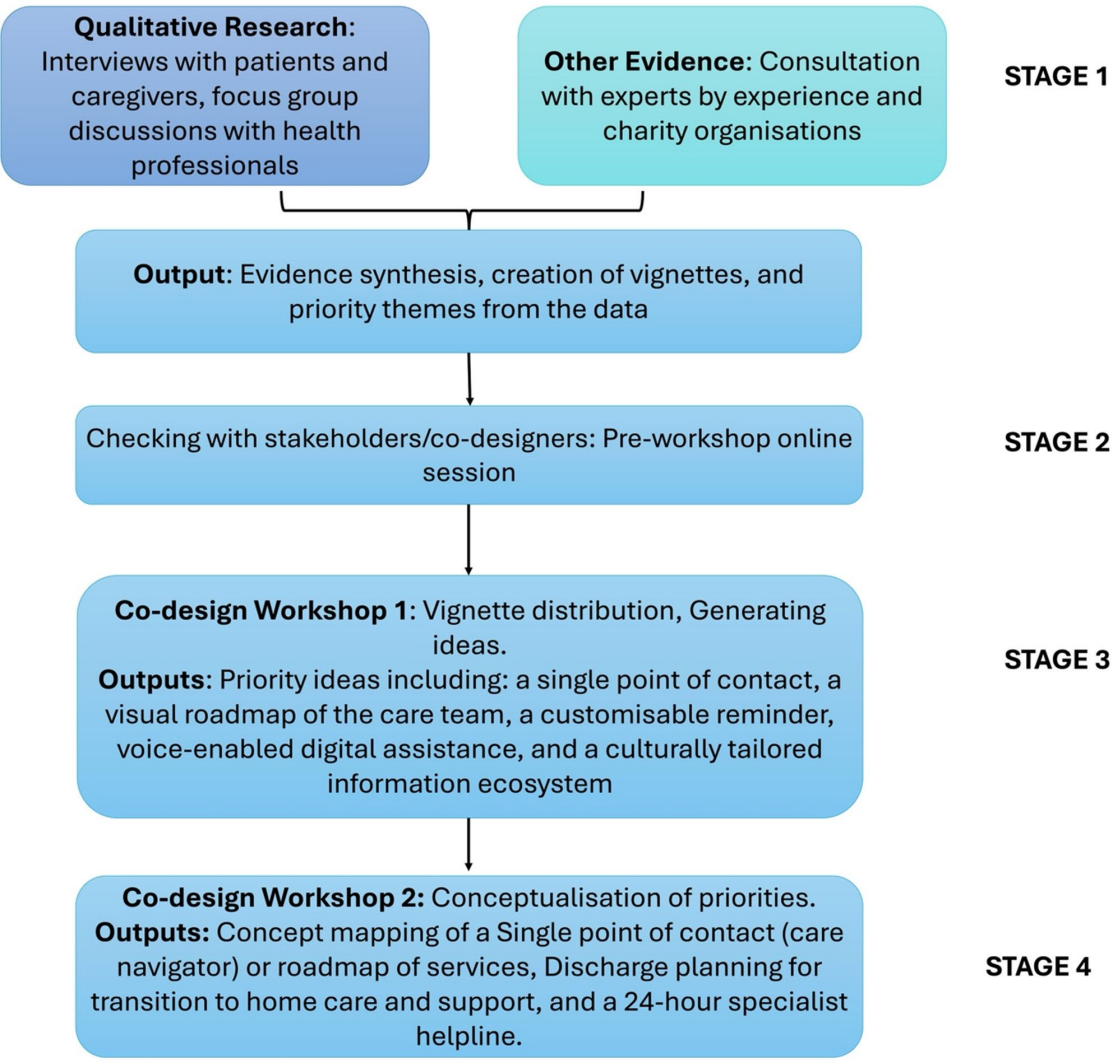
Overview of systematic sequential approach to intervention co-design

Figure 2 shows a summary overview of our systematic approach to the co-design process. After the workshop, the detailed notes taken, individual group concept map sheets and audio recordings of the discussions were collected. We analysed the workshop notes descriptively, focusing on the specific interventions proposed, potential challenges and evaluation strategies mentioned regarding those interventions. We integrated the analyses from Workshops 1 and 2 to map the problem areas identified through the co-designed interventions. Triangulation of perspectives (patients, caregivers, HCPs) across all workshops enabled us to ensure that the priorities identified were not merely individual concerns but were also shared by others.

In addition, we also documented the workshop process itself and gathered participant feedback about the co-design experience at the end of workshop 2 (for reflections on using this approach). Finally, throughout the co-design process, we adhered to participatory research quality criteria by practising reflexivity through regular team debriefs on power dynamics and engagement after each workshop, and by ensuring transparency through sharing the workshop documents on Google Drive and incorporating feedback from our PPI team member (V.B.), who also participated in the co-design process, on the contextualisation of the workshop findings.

Figure 2: Overview of systematic sequential approach to intervention co-design.

#### Outputs

Co-designers developed three actionable intervention proposals that collectively target gaps in support, care continuity and information accessibility. These were:

1. **Single Point of Contact (care navigator) or Roadmap of Services**

***Purpose and scope***. Recognising that patients frequently “*do not know whom to call*,” co-designers recommended a durable, fridge-mounted contact roadmap containing names, photographs, access hours, and one-line role descriptors for all relevant professionals, as well as a wallet-sized card indicating the single preferred point of contact.

*They might see a face but not necessarily know why they know that person*,* or they might have a problem and not know who to ring*. (HCP, Female < 65)

The benefits of a dedicated *care coordinator or navigator* role were emphasised strongly by participants. Co-designers envisioned that this coordinator could “*hold the roadmap*” of the patient’s journey, guiding them and troubleshooting issues (much like a case manager). This person (likely a specialist nurse or allied health professional) would be assigned to each patient at the time they are identified as having an incurable disease. Patient co-designers also liked the idea of *“one person who knows me”* in the system. A caregiver noted this would also ease their burden of chasing information.

***Infrastructure requirements***. HCP co-designers acknowledged that the care-navigator role could prevent many issues, but that it would require funding. For the fridge-mounted roadmap, participants felt that annual updating cycles, clear ownership for content maintenance, and parallel digital storage (e.g., via the clinical nurse specialist) would be necessary to keep information current and accessible if the hard copy were misplaced.

***Anticipated challenges***. In discussing this, some HCPs said that their service already allocates this role to a clinical nurse specialist. However, they admitted that workload and late referrals (only during the treatment phase) often made it challenging. The group’s solution was to formalise the role, so that when a patient is deemed to have incurable cancer, a coordinator is assigned (if not already done) and introduced. If the cancer centre’s nurses could not extend to post-treatment care, a palliative care nurse or even a trained navigator was an alternative. Concerns were also raised about information overload and unrealistic expectations of instant responses from HCPs when patient calls are made outside working hours. However, the provision of access hour labels and a brief “who to call when” infographic was recommended.

***Evaluation strategy***. The role’s effectiveness could be tracked using Patient-Reported Experience Measures (PREMs), which would capture the patient’s or caregiver’s perception of their care experience in aspects such as communication, timeliness, and others. The effectiveness of the roadmap can be evaluated based on how well and how long it has been used and retained in the home, as well as call volume analytics and tracking linked back to the helpline to determine whether queries are being appropriately channelled.

2. **Discharge Planning for Transition to Home Care and Support**

***Purpose and scope***. To help with transitions across care settings, the group advocated for a structured discharge meeting which includes both community and hospital staff, as well as the patient and caregiver, convened at least 48 h before the planned date of hospital discharge. Patients and caregiver co-designers stressed the importance of their own presence and that of allied health professionals at these meetings. This solution emerged to systematically fix the chaotic discharges that participants in the qualitative interviews described.

*We don’t tend to really get [occupational therapists] that involved in head and neck. But thinking about what you were just saying… the logistics of rearranging a room so that the plug socket for the suction machines is next to where the patient’s going to be sat*,* you’d really need the occupational therapist for that* (Co-designer-HCP Female, < 65)

*It’s a lot different learning how to handle all this equipment and medication in the confines of the hospital*,* [than] once we get home.* (Co-designer- Carer, Male, ≥ 65)

***Infrastructure requirements***. Participants considered that effective implementation would hinge on early notice from the medical team that discharge is imminent, and a template “holistic discharge summary” that goes beyond medical orders to include physiotherapy, dietetics, speech and language therapy (SALT), pharmacy, GP liaison, and palliative care arrangements. They envisioned a dedicated ‘Hospital to Home’ transition team to provide ongoing support and check-ins post-discharge.

***Anticipated challenges***. Three challenges were identified: Discharges on Fridays or weekends may risk delay because not all disciplines operate seven-day services; there is variation in community nurses’ training for complex airway and nutrition needs for this patient cohort; and the transfer of information to primary care is often relatively slow.

***Evaluation***. The proposed evaluation metrics for this solution were 30-day readmission rates, the timeliness of discharge summaries reaching primary care, and post-discharge satisfaction checks via phone calls or brief surveys to verify the adequacy of home support.

3. **24-Hour Specialist Helpline**

***Purpose and scope***. Participants stated that symptom crises usually occur at night, such as breathing difficulties, bleeding, and uncontrolled pain. They therefore envisioned a round-the-clock telephone (and SMS) helpline staffed by professionals with head and neck cancer expertise as the most immediate way to reduce anxiety, avert unnecessary emergency attendances, and normalise timely help seeking.

*Sometimes patients and relatives might downplay what they’ve understood… because they don’t want to feel as though they’re a burden. So*,* having one go-to person who knows my case rather than always calling a random clinic nurse would be great* (Co-designer-Patient, Male, ≥ 65)

*It would give you peace of mind knowing that someone is only a phone call away*,* even if it’s 3 in the morning* (Co-designer-Patient, Male, < 65)

***Infrastructure requirements***. Participants itemised: (i) a workforce model in which a dedicated rota is supplemented by a backup pool of staff to preserve continuity during sickness or annual leave; (ii) a multimodal access system with IT integration that allows real-time access to electronic records, with voice plus text capability for people rendered non-verbal post-laryngectomy (iii) a queue rather than voice mail system so calls are never abandoned; and (iv) a national rather than hospital specific footprint to pool staffing and avoid regional inequities. A searchable “clinical image map” of common post-treatment signs and symptoms was suggested as a clinical decision aid to guide triage and referral for the call handlers.

***Anticipated challenges***. The group acknowledged risks centred around the potential for: a single caller holding up other callers; patients calling from outside the regional area being excluded, if it’s not national; some patients from socio-economically deprived areas not engaging with the system; and the sustainability of funding. A suggested solution for mitigating the funding issues that might arise from such an intervention was to review the pilot phase analytics to evidence the demand for the resource before permanently commissioning it.

***Evaluation strategy***. The metrics used to assess whether this intervention is working could include call volume analytics (reason, duration, and clinical outcome); linkage of call data to subsequent healthcare use; and regular administration of patient-reported experience measures (PREMs) to identify high-frequency issues and system bottlenecks.

## Discussion

### Principal findings

This study describes using a co-design process to identify the most important priorities for patients with incurable HNC, generate and prioritise potential solutions and begin to design interventions to improve their care. The co-design workshops provided a clear set of priority domains for improving care, as well as a set of evidence and experience-informed interventions co-designed by patients, caregivers, and HCPs. The three prioritised interventions were a single point of contact (a dedicated care navigator or a roadmap to services), multidisciplinary discharge planning and support that included the patient and carer dyad, and a 24-hour specialist helpline.

Consistent with the findings of previous studies, interview participants (from our WP1) expressed a desire for multiple forms of information and support [32–34], a finding that resonated with our co-designers. Co-designers stated that patients and caregivers might feel unable to ask for information due to “*fear of being a burden to healthcare professionals”*. This may present a particular challenge for HNC patients, who often have specific conditions that make issues related to communication and swallowing more complex. The dedicated ‘care navigator/single point of contact’ role could help to address this problem. This role is well-established and has been adopted across various countries, disciplines, and healthcare systems (including the UK’s National Health Service (NHS) where it has proven effective in improving patient satisfaction [35], ensuring timely care, and enhancing treatment adherence [36, 37]. As another example, in the Expert Centre case within a Dutch hospital, a specialist nurse serving as a dedicated contact point for effective and efficient communication between patients and other HCP led to measurable improvement in patients’ perceived care quality [38]. Therefore, incorporating this role into HNC care has the potential to provide streamlined communication and better support for patients as they navigate their treatment needs and experiences.

As the care trajectories for patients with incurable HNC are inherently complex, involving interactions across numerous healthcare services and organisations [39], these patients routinely interact with multiple HCPs. WP1 showed that communication across these different services may be fragmented. Such fragmentation, particularly for patients with advanced cancer, can contribute to the increasing demands and stress-related issues for patients and caregivers [32], and is associated with preventable hospital admissions in the final phase of life [40]. The HCPs in the co-design workshops also acknowledged that current hospital discharges often focus on acute medical issues and may overlook the holistic needs of patients at home. This can leave patients and caregivers feeling unprepared for the transition to home care and can be equally overwhelming for patients already struggling with a complex situation [41]. To address this, it has been previously suggested that integrating a comprehensive discharge planning process at the point of hospital discharge [42], which involves the community health team and addresses the needs of this patient cohort and their caregivers at home, is crucial [43] and could potentially be more cost-effective for the healthcare system in the long run [44].

A 24-hour specialist phone support for people with incurable head and neck cancer (HNC) is the third priority intervention identified by our co-designers. This is also not a new system, and there is evidence that cancer helplines can improve symptom control and patient experience; a meta-analysis published in 2024 showed a moderate decrease in pain, fatigue, and depression from nurse-led telephone triage [45]. Observational studies have also shown that such services can lead to fewer unplanned hospital admissions and reduced treatment costs [46]. A recent palliative care trial showed that 24-hour telephone follow-up effectively addressed pain for nearly 40% of callers [47]. While our evidence primarily comes from mixed-cancer cohorts, qualitative findings suggest that patients with incurable diseases value immediate access to empathic and knowledgeable staff [48, 49], which are needs echoed by our co-designers. In practice, round-the-clock coverage demands a robust roster, plus contingencies for unexpected absences, to reduce service downtime and caller abandonment rates [50]. Our co-designers were also concerned that a single caller could monopolise the line, and that there could be geographic and socio-economic disparities, with rural and socio-economically deprived patients less likely to engage with virtual care [51, 52]. Moreover, to implement an intervention like this, early call-centre research recommends the use of real-time queue dashboards, call-back options, and time-limited consultations to balance access and therapeutic rapport [53].

Building on these findings, our co-designers corroborate a growing evidence base that 24-hour, specialist helplines may be a way to effectively address the complex and fluctuating needs of patients with incurable HNC. Successful implementation, however, will depend on sustained funding, a specialist workforce with capacity, validated decision-support algorithms, resilient queue management, and safeguards to ensure equity. Future research would need to assess the clinical and economic impact, while exploring how new (and pre-existing) non-verbal communications (digital) channels may mediate acceptability and access.

While the co-designed interventions may have broader relevance across cancer populations, participants emphasised that their importance is especially significant for individuals living with incurable HNC due to the rapid and severe impact it has on communication, swallowing, and breathing. The impact on speech, eating and breathing from tumours and their treatment within the head and neck region can significantly decrease a patient’s quality of life, contribute to psychological distress and make it difficult for them to interact with healthcare services [54]. The physical and functional impairment from these effects can impede timely help-seeking, symptom reporting, and care coordination, and are associated with greater social isolation and increased utilisation of emergency services, particularly as the disease progresses and the symptoms worsen [55].

### Our intervention development approach

To our knowledge, this is the first study to use a combination of four different activities (vignettes, prioritisation exercises, group concept mapping and facilitated group discussions) in co-design workshops focused on incurable HNC care needs. The prioritisation activities were an essential component of our co-design workshops, as they identified the most important areas for improvement from the participants’ perspectives. These activities, common in design-thinking workshops, engage participants in consensus-building and ranking exercises to pinpoint key issues that need to be addressed [56–58]. We used a ranking exercise, specifically dot-voting [30], to not only maintain focus on the specific ideas that were generated in each workshop but also enable all participants to contribute equally, without one or more confident voices dominating.

Other co-design studies with HNC patients have used ‘trigger films’ as video vignettes [1, 34] and, while we recognise the emotional and visual impact of this approach [59, 60], we chose to use written vignettes. We considered that written vignettes offered narrative depth [61] to patients’ (and caregivers’) experiences [62]. Additionally, because creating a video vignette or trigger film is ethically complex [63], resource-intensive, time-consuming, and requires substantial technical expertise [64, 65], a written vignette was considered a more practical and appealing option.

The last two activities conducted during the workshops facilitated concept mapping and group discussions to further develop these concepts using supporting materials. Both activities have been used individually in various patient experiences [66] and cancer studies [67–69], including in HNC [70], and they have individual strengths and weaknesses. For example, Alolayan (2023) used concept mapping to identify priorities for HNC treatment, reporting that while group methods can enhance engagement, there are challenges in managing diverse opinions [70].

Similarly, Gray et al. (2024) and McCaffrey et al. (2019) note that the structured approach of group concept mapping (GCM) effectively captures diverse perspectives and reduces researcher bias. However, they caution that some participants may not fully engage, regional viewpoints can vary, and it doesn’t allow for the deeper, back-and-forth conversations that uncover richer insights, such as facilitated discussions [66, 67]. Nevertheless, by combining both methods in our workshops, we were able to leverage their complementary strengths (the systemic nature and inclusivity of GCM and the relational dynamics and contextual insights of facilitated discussions), thereby enhancing the overall effectiveness of the co-design process.

While the four different activities used in this study may not always be applicable in their entirety to co-design workshops for other health conditions or populations, they enhance the evidence base on how to undertake co-design by providing practical insights likely to be of value to other researchers of HCPs embarking on this process.

### Strengths and limitations

A major strength of our study was that by using a sequential design, the interventions developed in the workshops were specifically designed to address the identified needs and preferences of HNC patients and their caregivers, who represent a particularly underserved patient group. This approach increases the likelihood of successful implementation and acceptance of the proposed interventions within clinical settings [1].

Although the intervention ideas were developed to address challenges faced by people with incurable HNC, many have wider relevance. This is because, within the NHS, patients with both curable and incurable diseases follow largely similar pathways and are supported by the same multidisciplinary teams; therefore, navigation difficulties, unclear points of contact, and fragmented communication between services will be common across the broader HNC population. While the use of vignettes grounded the conversations in the realities of an incurable disease, the natural solutions developed by the co-designers had relevance beyond the immediate scope of incurable HNC, highlighting one of the strengths of this work and illustrating the possible application of these interventions to a broader spectrum of patients.

Another strength of our co-design process, following Johnson et al.’s (2021) recommendations (for opportunities for emotional support for public members engaged in potentially demanding research [71], was that we ensured that participants were given adequate resources and support during the co-design workshops. That said, we acknowledge that these initiatives may not entirely mitigate the likelihood of some participants experiencing strong emotions due to the sensitive nature of HNC. Therefore, an ongoing evaluation of the co-designers’ well-being and feedback on areas of support provided is crucial for refining the co-design process in other studies of a similarly sensitive nature.

A limitation of our study, which has also been reported in experience-based co-design (EBCD) studies on HNC [1, 34], was that only a small number of HCPs participated in the workshops, possibly because these were conducted during working hours. Although we could fund their travel expenses and participation costs, we couldn’t cover the backfill of their clinical caseloads or any other work commitments they might have had at that time. Future workshops might consider flexible scheduling options or virtual participation techniques to increase participation and input from all relevant HCPs. However, while virtual participation offers accessibility and convenience, it has been shown to present several challenges in the co-design process. For example, Istanboulian et al. (2023) report that it’s harder to perform hands-on activities, some participants might not attend regularly, and organising co-design sessions could be a logistical burden [72]. Similarly, Sanders and Shen (2025) also note that it can be challenging to read verbal cues, and increased participant fatigue necessitates a more structured facilitation style to keep everyone engaged [73].

Another potential limitation was that although all patient co-designers participating in the workshops had a diagnosis of head and neck cancer, detailed clinical information, such as cancer stage, subtype, or prognosis, was not collected from them. This was a deliberate decision to avoid causing distress, maintain a supportive/collaborative environment, and keep the focus on service improvement rather than clinical disclosure. Consequently, not all patient co-designers involved may have been living with an incurable disease. However, the vignettes used in the workshops were developed from the interview dataset, which comprised only participants with a confirmed incurable head and neck cancer diagnosis. As such, while co-design participants were not required to have an incurable disease, the vignette content itself remained grounded in the experiences of this population. The workshops were solely intended to develop potential service interventions and co-design literature supports, including individuals across different illness trajectories, to broaden the scope and the relevance of the ideas generated [74–76]. The approach emphasises diverse perspectives, shared power, and inclusivity to foster richer idea generation and ensure that future service improvements reflect a wide range of user experiences [13, 29, 77].

Additionally, although we extended invitations to individuals with incurable HNC from three different regions in England, there was limited ethnic variation among those who participated. We recommend that future research should explore how the design and findings might be received or adapted across more diverse demographic and disciplinary contexts involving patients with incurable HNC.

## Conclusion

This work demonstrates how a co-design process can offer a valuable framework for generating solutions that have the potential to improve the care experience of patients living with HNC. By actively involving patients, caregivers, HCPs and researchers, the study identifies critical areas for improvement and encourages a more holistic approach to care that prioritises patient needs and preferences. Together, the interventions described by the co-design participants create a coherent service ecosystem: a 24-hour helpline as a ‘real-time’ safety net, a discharge MDT that brokers community handover, and a physical ‘roadmap’ (or care navigator) that anchors the care network in the patient’s own home.

Additionally, our use of vignettes, prioritisation exercises, and group discussions in the workshops facilitated meaningful conversations among co-designers, providing valuable insights into the specific challenges faced by this population, ultimately leading to the development of targeted interventions. Based on the success of these techniques and activities used during our co-design process, we recommend further exploration of these techniques to enhance patient engagement and co-design efforts across various healthcare contexts.

Finally, it is important to recognise that these proposed interventions may have a wider applicability across the HNC survivorship pathway; however, they are especially important for individuals with incurable HNC, whose rapidly changing clinical needs and increased dependence on multiple services exacerbate the impact of unclear navigation and fragmented communication between services. Building on these findings, our team has begun developing a prototype of the ‘roadmap to services’ intervention, with further co-refinement and feasibility testing planned. Therefore, the outputs of this study offer insight into the barriers in the current care pathway, highlighting key areas for improving continuity, access, and support within this vulnerable group, and laying the foundation for specific, patient-driven improvements to future HNC service delivery.

## Data Availability

Additional data is available from the corresponding author on reasonable request.

## Abbreviations

CNS: Clinical Nurse Specialist
EBCD: Experience-Based Co-Design
FGD: Focus Group Discussion
GCM: Group Concept Mapping
GP: General Practitioners
HCPs: Healthcare Professionals
HNC: Head and Neck Cancer
MDT: Multi-Disciplinary Team
NHS: National Health Service
NOK: Next of Kin
PPI: Patient and Public Involvement
PROMs/PREMs: Patient Reported Outcome/Experience Measures
SALT: Speech and Language Therapist
WP: Work Package
